# Draft genome of a multi-drug, metal and biocide-resistant strain of *Klebsiella variicola* isolated from the Yaque del Sur River in the Dominican Republic

**DOI:** 10.1128/mra.00700-25

**Published:** 2025-10-24

**Authors:** David Tavares Martins, Oscar Victor Cardenas Alegria, Thais Azevedo Cardoso, Antônio Márcio Gomes Martins-Júnior, Luiz Orlando Maroto-Martin, Edian Franco Franklin de Los Santos, Rommel Thiago Jucá Ramos

**Affiliations:** 1 Laboratory of Bioinformatic and Genomics of Microorganisms, Institute of Biological Sciences, Federal University of Pará-UFPA 37871, Belém, Pará, Brazil; 2 Centro Paraense de Computação Distribuída e de Alto Desempenho, Federal University of Para 37871, Belém, Pará, Brazil; 3 Institute of Biological Sciences, Universidade Federal de Minas Gerais, Belo Horizonte, Minas Gerais, Brazil; 4 Laboratório de Genética, Evolução e Bioinformática, Instituto Federal do Pará-IFPA 176849 , Tucuruí, Pará, Brazil; 5 Laboratory of Applied Biology and Bioinformatics, Department of Basic and Environmental Sciences, Instituto Tecnologico de Santo Domingo (INTEC) 37543, Santo Domingo, Dominican Republic; 6 Universidad Tecnológica de Santiago, Campus Central de Herrera 370455, Santo Domingo, Dominican Republic; 7 Instituto de Innovaccion en Biotecnologia e Industria (IIBI), Santo Domingo, Distrito Nacional, Dominican Republic; Wellesley College, Wellesley, Massachusetts, USA

**Keywords:** *Klebsiella variicola*, resistance, multi-drug, Caribbean

## Abstract

*Klebsiella variicola* strain YSP4_INTEC was isolated from a Dominican Republic River and had its genome sequenced (size: 5,611,899 bp and GC content: 57.19%). The genomic analysis identified various genes involved in plant colonization mechanisms, plus a wide array of multi-drug, biocide, and multi-metal resistance genes.

## ANNOUNCEMENT

*Klebsiella variicola* is a highly adaptable bacterial species found in diverse environments and maintains complex mutualistic or parasitic relationships with plants, fungi, or animals (1). It exhibits plant growth-promoting genes and can remove heavy metals and phenolic compounds from the environment (2–5). However, some strains are highly virulent and resistant to antibiotics, causing severe infections in humans (6–8). Due to limitations in automated microbiology equipment, differentiating *Klebsiella pneumoniae* from *K. variicola* remains challenging (2). This difficulty highlights the need for genomic studies, especially in places like the Dominican Republic, where data on related infections are scarce.

River water samples were collected from Tamayo, Bahoruco (18°23.962′N, 71°11.165′W). The water was serially diluted (10^−1^ to 10^−4^) and plated on MacConkey Agar, incubated at 37°C for 24 h. Colonies were isolated on the same medium and cultured on CHROMagar Orientation. The COL-APSE and SuperCARB culture media were employed to identify colistin-resistant and carbapenem-resistant colonies, respectively. The DNA was extracted from a single colony with the DNeasy Blood and Tissue (QIAGEN) Kit and quantified with a Qubit fluorometer. Sequencing was performed by the NovoGene company and conducted in the Illumina NovaSeq6000 platform with a microbial whole genome library (2 × 150 bp paired-end) without customizations. Quality control was conducted using AdapterRemoval (3.0.0) (9), KmerStream (1.1) (10), and FastQC (0.12.1) (11). Genome assembly utilized Edena (3.131028) (12), SPAdes (4.2.0) (13), and Unicycler (0.5.1) (14), with the best assembly selected based on QUAST (5.3.0) (15) results. Annotation was performed using Prokka (1.14.6) (16), and MOB-recon (3.1.9) (17) was used for plasmid annotation and extraction. These steps were performed via the Assembly Hi-Seq pipeline (18). Taxonomic classification was followed using GTDB-Tk v2 (v1.6.0) (19). Annotation was performed using PATRIC (https://www.bv-brc.org/) (20), and AMR identification was performed with AMR++ (v2.0.2) (21) and CARD (v6.0.6) (22). VFAnalyzer (https://www.mgc.ac.cn/VFs/main.htm) (23) was used for virulence gene identification. CheckM (v1.0.18) (24) was used for completeness evaluation. Phylogenetic trees were reconstructed using a maximum likelihood criterion in RaxML-NG, comparing (25) the isolate to 19 *Klebsiella* species genomes from NCBI. All the tools were used with default parameters.

**Fig 1:**
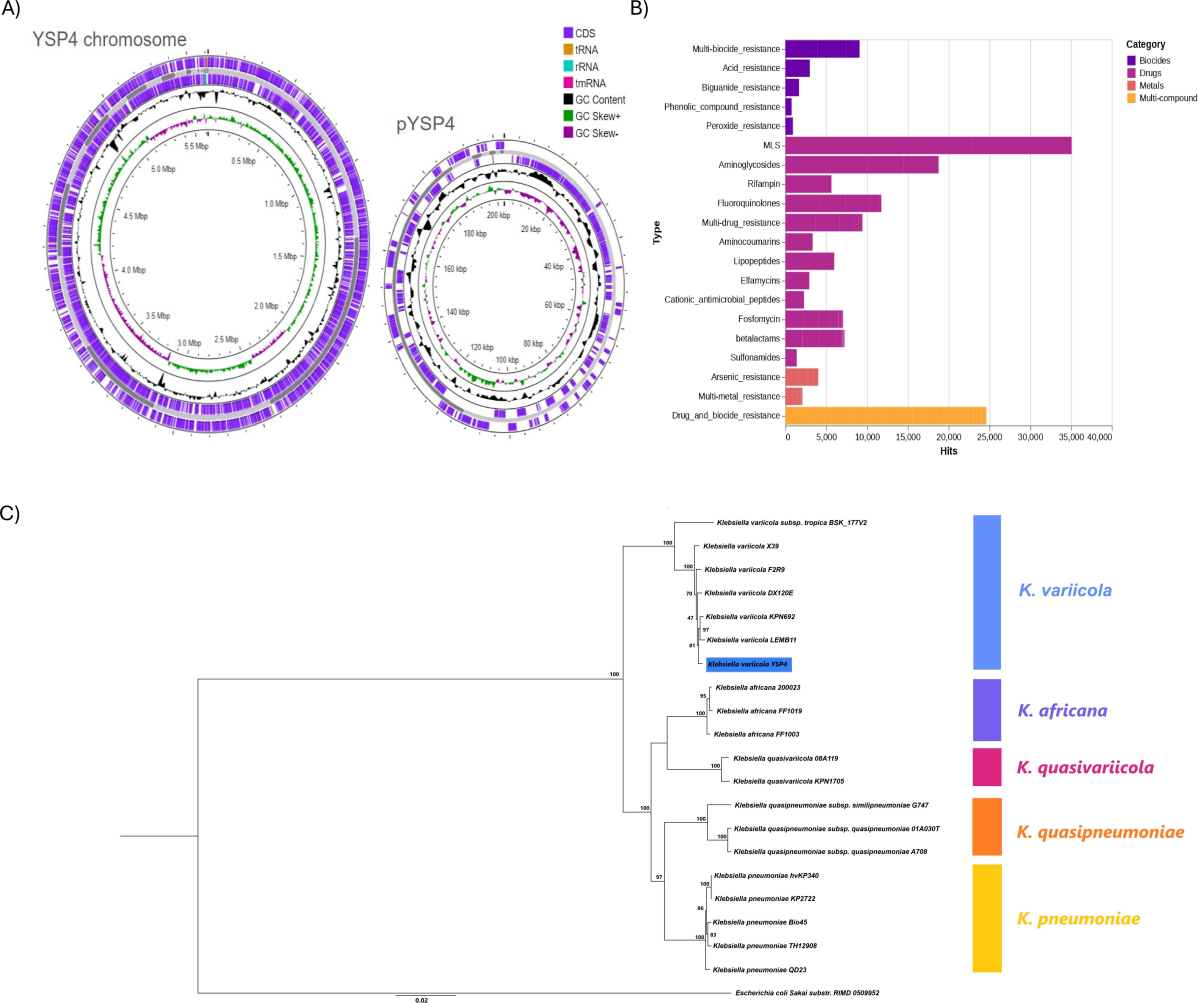
(**A**) Circular map of chromosome and plasmid from *K. variicola* strain YSP4_INTEC’s annotated. From outside to the center: genes on forward strand, genes on reverse strand, GC content, and GC skew. Circular map was generated by Proksee. (**B**) Reads of YSP4_INTEC’s sequencing distribution over four different categories of bacterial resistance genes in the MEGARES database analyzed by AMR plus plus. (**C**) Phylogenetic tree of the *Klebsiella* group genome sequences showing the position of *K. variicola* strain YSP4_INTEC. *Escherichia coli* Sakai substr. RIMD 0509952 is used as an outgroup. The values located in front or behind the tree nodes represent the bootstrap support value.

The analysis revealed a 5,611,899 bp chromosome assembled in 60 contigs (N50 = 715,876 bp, L50 = 3, and GC content = 57.19%) with 228× coverage, 99.61% completeness, and 9,690,956 total reads. The genome harbored 5,796 coding sequences (CDSs), 76 tRNAs, and 5 rRNAs. A total of 4,835 CDSs were annotated with known functions, and 961 were hypothetical proteins. The plasmid was assembled in 8 contigs displaying 202,973 bp (N50 = 113,706 bp) and harbored 120 protein-coding genes and 154 hypothetical proteins. Phylogenetic analysis placed the isolate within the *K. variicola* clade with 100% bootstrap support (Fig. 1).

The bacterium harbors 27 putative antibiotic and biocide resistance genes, including multi-drug efflux pumps, as well as arsenic and multi-metal resistance genes. It contains 63 putative virulence genes distributed in adherence, iron uptake, secretion systems, and proteases, including a protease involved in the pathogenesis of *Yersinia pestis* (26). The strain also carries genes involved in nitrogen fixation, phosphate solubilization, and phytohormone production typical in plant-interacting bacteria. The presence of genes associated with plant colonization and biocide resistance substantiates the possibility of pathogen transmission through plants to animals and humans.

## Data Availability

This Whole Genome Shotgun project has been deposited in GenBank under the bioproject PRJNA951268 and biosample SAMN49305619. The raw data are available at SRR34021923 and the assembly at GCF_051107315.1. Supplementary information can be found in 10.5281/zenodo.17019365.
